# Metagenomic insights into *Heimdallarchaeia* clades from the deep-sea cold seep and hydrothermal vent

**DOI:** 10.1186/s40793-024-00585-2

**Published:** 2024-06-22

**Authors:** Rui Liu, Ruining Cai, Minxiao Wang, Jing Zhang, Huan Zhang, Chaolun Li, Chaomin Sun

**Affiliations:** 1 CAS and Shandong Province Key Laboratory of Experimental Marine Biology, Institute of Oceanology, Chinese Academy of Sciences, Qingdao, China; 2 Laboratory for Marine Biology and Biotechnology, Qingdao Marine Science and Technology Center, Qingdao, China; 3 Center of Deep Sea Research, Institute of Oceanology, Chinese Academy of Sciences, Qingdao, China; 4 Center of Ocean Mega-Science, Chinese Academy of Sciences, Qingdao, China

**Keywords:** *Heimdallarchaeia*, Cold seep, Hydrothermal vent, Metabolism pathway, Light-sensing, Microoxic lifestyle

## Abstract

*Heimdallarchaeia* is a class of the *Asgardarchaeota*, are the most probable candidates for the archaeal protoeukaryote ancestor that have been identified to date. However, little is known about their life habits regardless of their ubiquitous distribution in diverse habitats, which is especially true for *Heimdallarchaeia* from deep-sea environments. In this study, we obtained 13 metagenome-assembled genomes (MAGs) of *Heimdallarchaeia* from the deep-sea cold seep and hydrothermal vent. These MAGs belonged to orders *o*_*Heimdallarchaeales* and *o*_*JABLTI01*, and most of them (9 MAGs) come from the family *f*_*Heimdallarchaeaceae* according to genome taxonomy database (GTDB). These are enriched for common eukaryote-specific signatures. Our results show that these *Heimdallarchaeia* have the metabolic potential to reduce sulfate (assimilatory) and nitrate (dissimilatory) to sulfide and ammonia, respectively, suggesting a previously unappreciated role in biogeochemical cycling. Furthermore, we find that they could perform both TCA and rTCA pathways coupled with pyruvate metabolism for energy conservation, fix CO_2_ and generate organic compounds through an atypical Wood-Ljungdahl pathway. In addition, many genes closely associated with bacteriochlorophyll and carotenoid biosynthesis, and oxygen-dependent metabolic pathways are identified in these *Heimdallarchaeia* MAGs, suggesting a potential light-utilization by pigments and microoxic lifestyle. Taken together, our results indicate that *Heimdallarchaeia* possess a mixotrophic lifestyle, which may give them more flexibility to adapt to the harsh deep-sea conditions.

**Supplementary Information:**

The online version contains supplementary material available at 10.1186/s40793-024-00585-2.

## Introduction

Archaea are important microorganisms that play important roles in the biogeochemical cycle of Earth [1], and are indispensable for the study of evolution [2]. To date, four supergroups of archaea have been described: Euryarchaeota, TACK, Asgard, and DPANN [3]. Breakthroughs in metagenomic sequencing technology are rapidly transforming our understanding of microbial evolution, particularly with the discovery of the *Asgardarchaeota* phylum and the prediction of their position at the base of the eukaryotic tree of life [4]. The *Heimdallarchaeia* as well as their newly derived orders (including *o_Hodarchaeales*, *o_Heimdallarchaeales*, and *o_JABLTI01* according to genome taxonomy database, GTDB) [5] currently represent the closest predicted archaeal relatives of eukaryotes [6]. Although they have been given a new name to *o*_*JABLTI01* as *Gerdarchaeales* in recent literature [7], the original names are still used here in order to be consistent with the GTDB (R220 taxonomy from v2.4.0). Compared with other members of the *Asgardarchaeota* (e.g. *Lokiarchaeia* [8] and *Thorarchaeia* [9]), studies of *Heimdallarchaeia* are lagging due to a lack of metagenomic data and cultured strains. Nonetheless, the available genomic information supports the hypothesis that *Heimdallarchaeia* could survive in strictly anaerobic habitats, as well as in a sunlit microoxic niche [10]. The aerobic respiration would allow *Heimdallarchaeia* to use a wide range of organic substrates [8], and enable them to oxidize organic substrates by using oxygen as an electron acceptor, and allow them to conserve the energy by coupling ferredoxin reoxidation to respiratory proton reduction [11].

Metagenome-assembled genomes (MAGs) of *Heimdallarchaeia* class as well as their new derived clades were obtained from both marine and fresh water environments [4, 10, 12]. But the number of *Heimdallarchaeia* MAGs was much lower than *Lokiarchaeia* and *Thorarchaeia* in the database, which greatly limits the understanding of their metabolism, lifestyle, and contributions to biogeochemical cycling. Previous studies have mentioned that *Heimdallarchaeia* clades, including *o*_*JABLTI01* and *o*_*Hodarchaeales* possessed citrate cycle (TCA), succinate dehydrogenase and NADH-quinone oxidoreductase for aerobic respiration, and rhodopsins for light sensing [2, 10]. These unique archaea may have existed in microoxic and light-exposed habitats during their evolutionary history [4, 10, 13, 14]. However, many key metabolism pathways were indicated to be lacking in MAGs of *Heimdallarchaeia*, such as sulfur metabolism, the Wood-Ljungdahl (WL) pathway, and the reductive citrate cycle (rTCA) [2, 6, 15]. These findings suggest that the metabolism and lifestyle of *Heimdallarchaeia* remains to be explored.

Cold seeps and hydrothermal vents are two special deep-sea environments rich in methane, carbon dioxide (CO_2_), and sulfur. These are ideal locations for the study of biogeochemical cycling, novel metabolic pathways, and the biological origins and evolution of life [16–19]. Sulfur metabolism and CO_2_ fixation are thought to be important metabolic pathways for microorganisms in these environments [20–24]. It is generally believed that there is no light in the deep-sea environment below 1,000 m. However, the geothermal light has been mentioned in hydrothermal vents to provide a selective advantage for the evolution of photosynthesis from a chemotrophic microbial ancestor [25]. In addition, some bacteria have been reported to use light-sensing molecules for phototaxis toward light associated with the geothermal light in deep-sea vents [25–28]. For archaea, key enzymes involved in photosynthetic pigment synthesis have been reported in *Thermoproteota* (former *Crenarchaeota* and *Bathyarchaeota*) [29, 30]. Therefore, we are interested in whether some photosynthetic pigments are also present in *Asgardarchaeota* from deep-sea vents.

In the present study, we first analyzed the community structure of archaea in deep-sea cold seep and vent sediment, and then obtained 13 MAGs of *Heimdallarchaeia*, belonging to order *o*_*Heimdallarchaeales* (*o*_*UBA460*) and *o*_*JABLTI01*, respectively. Based on these MAGs, we confirmed the eukaryotic signatures of deep-sea *Heimdallarchaeia*; identified indications for their involvement in sulfur, nitrogen, and carbon cycling; and discovered their potential aerobic light sensing lifestyle. This work could pave the way for the future explorations of unexpected light utilization mechanisms and other special environmental adaptations possessed by *Heimdallarchaeia* in the deep biosphere.

## Results and discussion

### Phylogenetic status and eukaryotic signatures of deep-sea *Heimdallarchaeia*

**Fig. 1:**
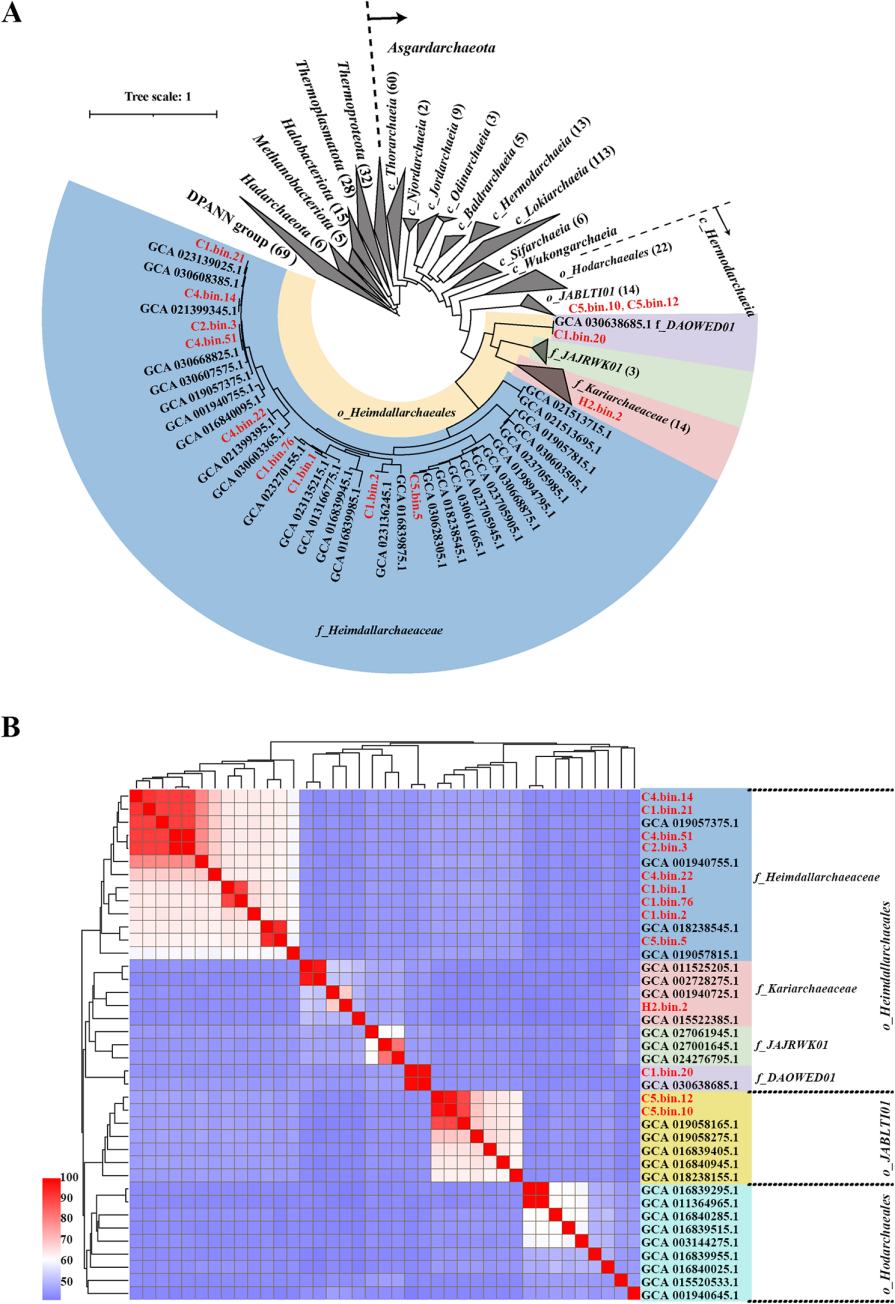
Phylogenetic analysis of *Heimdallarchaeia* clades. (**A**) Maximum-likelihood phylogeny of *Heimdallarchaeia* MAGs. Phylogenetic analysis was performed based on concatenation of 53 marker proteins for Archaea, which were chosen by Phylosift (1,000 bootstrap replicates). Detailed sequence information from different species in compressed clades is listed in Supplementary Dataset 1. (**B**) Amino acid identity correlation matrix of MAGs of *Heimdallarchaeia* clades was calculated by Compare M

To investigate the metabolic characteristics of deep-sea *Heimdallarchaeia*, we sampled four sediments (C1, C2, C4, and C5) from a deep-sea cold seep (depth greater than 1,100 m) in the South China Sea, and one sample (H2) from a deep-sea hydrothermal vent (depth 2,194 m, outside of the black chimney, environmental temperature 5.5 °C) in the Western Pacific Ocean (Table S1). These environments are rich in CH_4_, sulfur, and different metal ions (Figure S1). Metagenomic DNA from these five samples was extracted and sequenced. Sequence statistics indicated that proportion of annotated genes belong to *Heimdallarchaeia* were relatively abundant among *Asgardarchaeota* in both cold seep and vent environments (Figure S2). To explore the metagenomic characteristics of these *Heimdallarchaeia*, 13 MAGs were obtained using a hybrid binning strategy combined with manual inspection and data curation. Seven of 13 MAGs (> 80% completeness, < 5% contamination) were considered as high-quality genomes according to the reported standards (Table S2) [31]. Other MAGs were of medium-quality (> 50% completeness, < 5% contamination) [32], except for C2.bin.3, C5.bin.12 and H2.bin.2. However, since the completeness of these three MAGs was higher than 70%, we also used them for subsequent functional gene annotation and analysis. The maximum-likelihood phylogenetic tree was generated based on concatenation of 53 marker proteins for Archaea from GTDB database (Release 220). Both the MAGs from the present study and other published *Heimdallarchaeia* MAGs clustered with *Asgardarchaeota* members, and displayed an obvious evolutionary distance from other archaeal phyla (Fig. 1A, Supplementary Dataset 1). These 13 MAGs fallen in two orders (*o*_*Heimdallarchaeales*, and *o*_*JABLT101*) based on the phylogenomic tree were further used for the amino acid identity (AAI) analysis (Fig. 1B) [6]. Among them, 11 MAGs mainly belonged to three families in *o*_*Heimdallarchaeales*, including *f*_*Heimdallarchaeaceae* (C1.bin.1, Ci.bin.2, C1.bin.21, Ci.bin.76, C2.bin.3, C4.bin.14, C4.bin.22, C4.bin.51 and C5.bin.5), *f*_*Kariarchaeaceae* (H2.bin.2) and *f*_*DAOWED01* (C1.bin.20). Of note, *f*_*Kariarchaeaceae*, *f*_*DAOWED01* and *f*_*JAJRWK01* were displayed a more similar identity distinguishing from *f*_*Heimdallarchaeaceae* MAGs in *o*_*Heimdallarchaeales* according the results both of phylogenetic tree and AAI analysis (Fig. 1). However, according to the analysis results of AAI, we found that the corresponding values of other three families (*f*_*Kariarchaeaceae*, *f*_*DAOWED01* and *f*_*JAJRWK01*) and *f*_*Heimdallarchaeaceae* ranged from 43 to 45%, but the values of *o*_*JABLTI01* and *f*_*Heimdallarchaeaceae* ranged from 46 to 47%, and the values of *o*_*Hodarchaeales* and *f*_*Heimdallarchaeaceae* ranged from 43 to 45% (Supplementary Dataset 1). This result might indicate that AAI analysis can only be used to define taxonomic ranks below the family level [31]. In addition, C1.bin.20 is the second reported MAG in *f*_*DAOWED01* according to records of the GTDB database. This will further enrich the species composition of *f*_*DAOWED01* family.

As the closest archaeal lineage to eukaryotes, eukaryote-specific proteins (ESPs) were indeed identified in the present 13 MAGs of *Heimdallarchaeia*, which is consistent with other members of the *Asgardarchaeota* (Figure S3, Supplementary Dataset 2). However, we found that the distribution of these ESPs was different in order levels of *Heimdallarchaeia* [6]. For instance, some ESPs, including eukaryotic ribosomal proteins, nucleus related proteins, vacuoles and signal transforming related proteins were found to have an almost complete distribution in all 13 MAGs (Figure S3, Supplementary Dataset 2). These ESPs were mainly involved in the storage of genetic material, gene transcription, protein translation, and material transport processes, which were recognized as the “basic part” of eukaryotic cells [10, 14, 33–35]. On the other hand, ESPs of ubiquitin-proteasome system, cytoskeleton, mitochondrion, and chloroplast, viewed as the “functional part” of eukaryotic cells, showed different distribution characteristics between *o*_*Heimdallarchaeales* and *o*_*JABLTI01* (Figure S3, Supplementary Dataset 2). Despite the lack of hard evidence, we speculate that the differences in order levels of *Heimdallarchaeia* might be related to the subsequent evolution of cellular complexity and functional differentiation of eukaryotic cells [7].

### *Heimdallarchaeia* clades could participate in the sulfur biogeochemical cycle

**Fig. 2:**
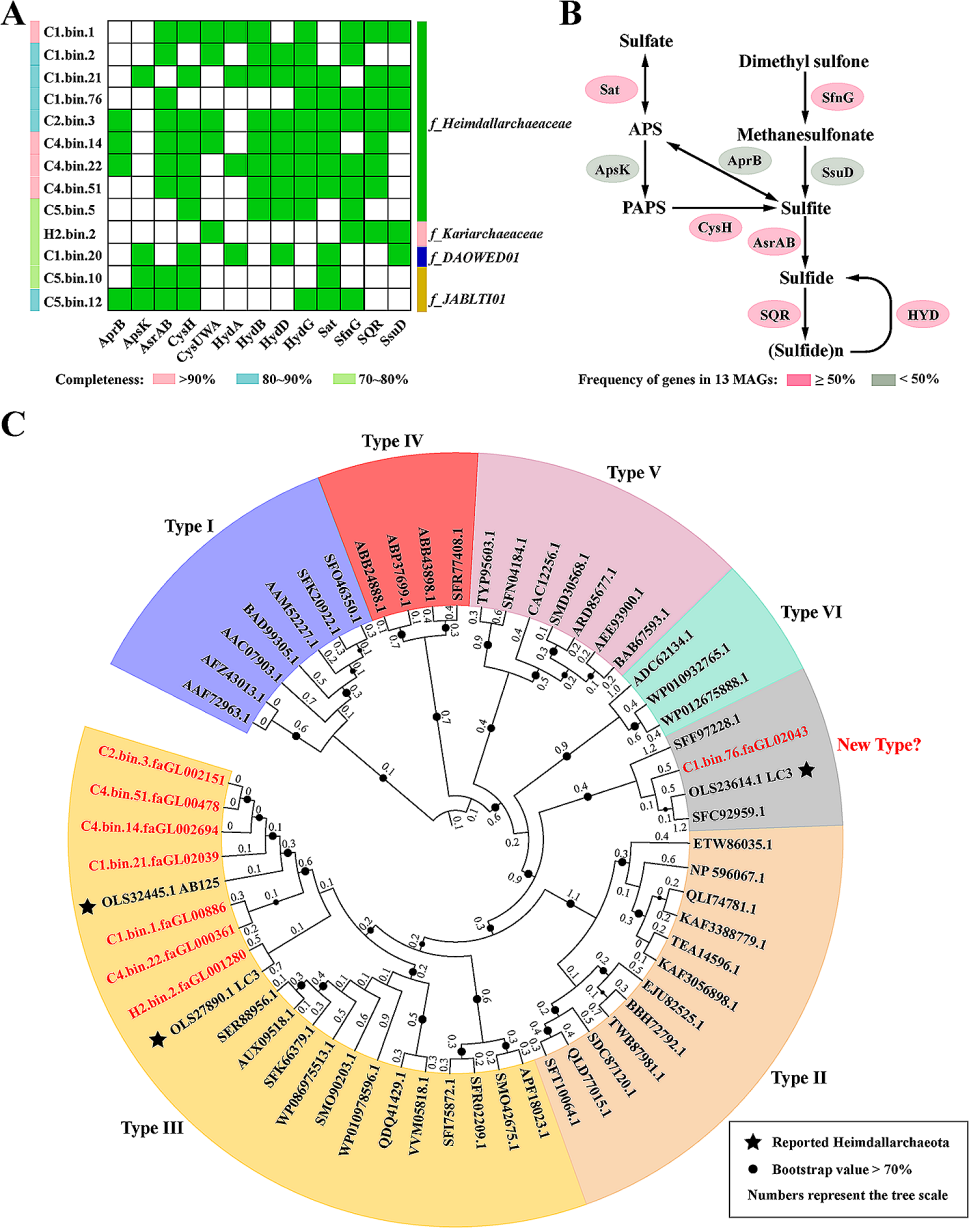
Sulfur metabolic pathway identified in *Heimdallarchaeia* MAGs. (**A**) Distribution of identified key enzymes involved in the sulfur metabolism of *Heimdallarchaeia* clades. The presence of enzymes involved in the sulfur metabolic pathway is indicated for each MAG using green colored rectangles. (**B**) Sulfur metabolic pathway identified in *Heimdallarchaeia* clades. (**C**) Maximum-likelihood phylogeny of sulfide: quinone oxidoreductases (SQRs) identified in *Heimdallarchaeia* clades (1,000 bootstrap replicates). Nodes indicate bootstrap values greater than 70. Numbers represent the tree scale of each branch. The previously reported SQRs in MAGs of *Heimdallarchaeia* were labeled by the black star. AprB, adenylylsulfate reductase, subunit B; ApsK (CysC), adenylyl-sulfate kinase; AsrA and AsrB, anaerobic sulfite reductases; CysH, phosphoadenosine phosphosulfate reductase; CysA, sulfate transport system ATP-binding protein; CysU, sulfate transport system permease protein; CysW, sulfate/thiosulfate transport system permease protein; HydA, HydB, HydD and HydG, sulfhydrogenases. Sat, sulfate adenylyltransferase; SfnG, dimethylsulfone monooxygenase; SQR, sulfide: quinone oxidoreductase; SsuD, alkanesulfonate monooxygenase; SoxB, S-sulfosulfanyl-L-cysteine sulfohydrolase; TST, thiosulfate/3-mercaptopyruvate sulfurtransferase. Detailed protein information related to this figure is listed in Supplementary Dataset 3

Sulfur cycling is believed to be a dominant form of metabolism for microorganisms living in the sampling locations presented in this study [36]. However, recent studies have shown that many key molecules of sulfur metabolic pathway are absent in the MAGs of *Heimdallarchaeia* and other *Asgardarchaeota*, such as dissimilatory sulfite reductase (DsrA), adenylylsulfate reductase (AprA) and anaerobic sulfite reductases (AsrAB) [2, 6, 11]. In this study, although DsrA and AprA proteins were also absent in our obtained MAGs, AprB and AsrAB instead could be identified from *Heimdallarchaeia*. In addition, other key enzymes in inorganic sulfur metabolism, including sulfate transport proteins (CysUWA), sulfate adenylyltransferase (Sat), adenylylsulfate kinase (ApsK), and phosphoadenosine phosphosulfate reductase (CysH), were widely distributed in the MAGs of the cold seep *Heimdallarchaeia* (Fig. 2A and B, Supplementary Dataset 3). Sulfate is known to be important environmental factors in the sulfate methane transition zone (SMTZ) of cold seeps [16, 37, 38]. Thus, our results suggest that *Heimdallarchaeia* inhabiting cold seep sediments have the potential to participate in inorganic sulfur metabolism for energy production through assimilatory and partly dissimilatory sulfate reduction pathways [39, 40]. In contrast, mostly enzymes (AprB, ApsK, CysH, and Sat) involved in sulfate and sulfite metabolism were absent in the MAG H2.bin.2 obtained from the vent environment. However, enzymes associated with dimethyl sulfone (DMS) metabolism, such as dimethyl sulfone monooxygenase (SfnG) and alkanesulfonate monooxygenase (SsuD), were identified (Fig. 2A and B, Supplementary Dataset 3), suggesting that some members of *Heimdallarchaeia* may be able to perform organic sulfur metabolism.

Notably, sulfide: quinone oxidoreductase (SQR) is broadly distributed in *Heimdallarchaeia* MAGs (Fig. 2A, Supplementary Dataset 3). SQR is known as one of the most widespread markers of marine sulfur-oxidizing microorganisms [41, 42]. It is a ubiquitous membrane-bound flavoprotein involved in sulfide detoxification via the oxidization of sulfide to zero-valent sulfur, through which electrons are transferred to the membrane quinone pool for energy conservation processes [42]. Previous studies have reported that *Heimdallarchaeia* could uniquely metabolize H_2_S [8, 10]. Therefore, SQR protein could play an important role in this metabolic process. In the present study, seven out of nine *f*_*Heimdallarchaeaceae* MAGs and H2.bin.2 contained complete amino acid sequences for SQR, most of which belonged to the type III group (Fig. 2C), consistent with SQRs identified in other archaea [41, 43, 44]. This ratio is significantly higher than the previously reported SQRs in *Heimdallarchaeia* MAGs [8]. An SQR present in MAG C1.bin.76 was found to form a distinct clade with SQRs from *Streptomyces aidingensis* (SFC92959.1), *Salegentibacter agarivorans* (SFF97228.1) and *Heimdallarchaeia* LC3 (OLS23614.1). This clade is distinct from the six typical groups (Fig. 2C), suggesting that a novel type of SQR group may exist.

Based on these results, we propose that more complete sulfur metabolism pathways (including assimilatory sulfate reduction and sulfide oxidation) in these deep-sea *Heimdallarchaeia* MAGs may be due to the higher concentrations of sulfur compounds in cold seep environments than previously reported *Asgardarchaeota* MAGs [2, 4, 11, 45, 46]. Therefore, *Heimdallarchaeia* clades may be important participants in sulfur cycling in the cold seep environments, particularly a higher concentration of sulfide (reach the greatest > 20 mM in the surface sediments) in deep-sea cold seeps [46].

### *Heimdallarchaeia* clades use diverse nitrogen compounds for growth

Nitrogen is the fourth most abundant element in cellular biomass, and it comprises the majority of Earth’s atmosphere [47, 48]. However, nitrogen is a limiting nutrient for biological systems in marine environments [49–51]. Hence, the nitrogen cycle is critical for both the growth of microorganisms and the biogeochemical cycles of the ocean [48–50]. Like sulfur metabolism pathways, the nitrogen metabolism pathways present in *Heimdallarchaeia* that derived from cold seeps and vents are different. The enzymes responsible for nitrate reduction to nitrite (NarI) [48], nitrite reduction to ammonium (NirD) [48, 49], nitrite reduction to nitric oxide (NirK and NirS) [52–54], hydroxylamine reduction to ammonia (Hcp) [48, 55], and ammonia transformation to glutamate (GlnA, GltD and GdhA) were all identified in the MAG H2.bin.2 obtained from vent sediment. However, these enzymes, with the exception of enzymes responsible for the transformation of ammonium to glutamate, were almost not detected in cold seep *Heimdallarchaeia* MAGs (Figures S4A and S4B, Supplementary Dataset 4). These results suggest that *Heimdallarchaeia* living in vents may play important roles in nitrate reduction, while those living in cold seeps may be essential participants in the metabolism of ammonia, which is potentially derived from the methylamine in this environment. Methylamine is thought to be an important nitrogen source for marine microorganisms, and is released through the biodegradation of proteins and N-containing osmolytes [56, 57]. In cold seep environments, methylamine is also a key substrate of archaeal methanogenesis, a process which may release a large amount of ammonia into the environment [58]. We consistently found large amounts of methane in cold seep sampling sites (Figure S1). Therefore, we infer that the different pathways for sulfur and nitrogen metabolism identified in deep-sea *Heimdallarchaeia* clades from diverse habitats may be the result of their long-term adaptation to the deep-sea extreme environment.

Nitrilase (nitrile aminohydrolase) has also been widely identified in deep-sea MAGs of *Heimdallarchaeia* clades (Figures S4A and S4B, Supplementary Dataset 4). Nitrilase catalyzes the hydrolysis of nitriles to form a carboxylic acid product with the concomitant release of ammonia [59, 60]. The existence of nitrilase in microorganisms endows them with the ability to use nitriles as a source of nitrogen for growth [61, 62]. Nitrilases have been divided into six subgroups according to their substrates (e.g. aliphatic and aromatic nitriles [59, 63] or amides [59, 64]). Phylogenetic analysis revealed that nitrilases in deep-sea MAGs of *Heimdallarchaeia* clades were mainly clustered in clades induced by aliphatic nitriles and amides (Figure S4C), suggesting that abundant aliphatic nitriles and amides might exist in the deep sea. We speculate that *Heimdallarchaeia* clades use diverse nitrogen compounds for growth, and play important roles in nitrogen cycling in deep-sea environments.

### *Heimdallarchaeia* sense the light by chlorophyll and carotenoid

**Fig. 3:**
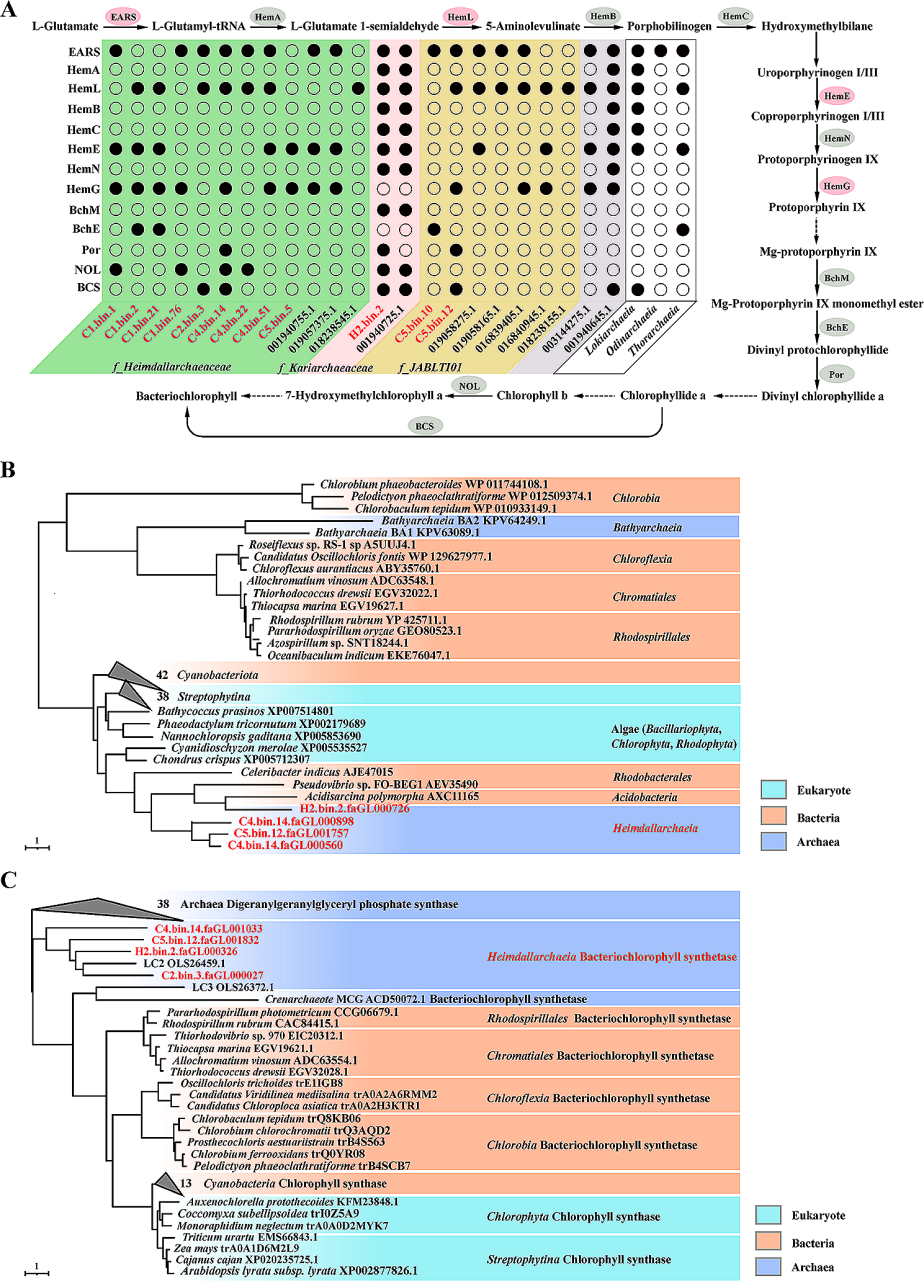
Porphyrin and bacteriochlorophyll biosynthesis pathways identified in *Heimdallarchaeia* MAGs. (**A**) Analysis of porphyrin and bacteriochlorophyll biosynthesis in different *Heimdallarchaeia* MAGs. (**B**-**C**) Phylogenetic analyses of protochlorophyllide reductase (Por) and bacteriochlorophyll synthetase (BCS). A rooted maximum-likelihood tree of Por (**B**) or BCS (**C**) homologs derived from different photosynthetic organisms identified in this work (1,000 bootstrap replicates). The solid arrows indicate the enzymes associated with bacteriochlorophyll biosynthesis present in *Heimdallarchaeia* MAGs. Dotted arrows indicate the enzymes associated with bacteriochlorophyll biosynthesis absent in MAGs. The gray box highlights MAGs from *o_Hodarchaeales*. The frame highlights assembled genomes of other Asgard archaea. The red highlights MAGs obtained in this study. EARS, glutamyl-tRNA synthetase; HemA, glutamyl-tRNA reductase; HemL, glutamate-1-semialdehyde 2,1-aminomutase; HemB, porphobilinogen synthase; HemC, hydroxymethylbilane synthase; HemE, uroporphyrinogen decarboxylase; HemN, coproporphyrinogen oxidase; HemG, protoporphyrinogen oxidase; HemH, protoporphyrin/coproporphyrin ferrochelatase; BchM, magnesium-protoporphyrin O-methyltransferase; BchE, anaerobic magnesium-protoporphyrin IX monomethyl ester cyclase. Por, protochlorophyllide reductase; NOL/NCY1, chlorophyll(ide) b reductase NOL/NCY1; BCS, bacteriochlorophyll synthase. The detailed information of key enzymes involved in bacteriochlorophyll biosynthesis and proteins used for phylogenetic analyses is listed in the Supplementary Dataset 5

Previous reports of the existence of rhodopsins in archaeal phyla (e.g. *Bathyarchaeia*, *Lokiarchaeia* and *Heimdallarchaeia* [10, 30]) have suggested that archaea can sense the light. In this study, no rhodopsin homologs were identified in the present MAGs of deep-sea *Heimdallarchaeia* clades. However, many typical chloroplastic proteins (including protochlorophyllide reductase, chlorophyll(ide) b reductases NOL/NYC1, NAD(P)H quinone oxidoreductase, and the photosystem I assembly proteins Ycf3 and phycocyanobilin lyase) were identified (Figure S3, Supplementary Dataset 2). Comparative genomic analysis revealed that a series of enzymes involved in the porphyrin and bacteriochlorophyll synthesis pathways were present in MAGs of deep-sea *Heimdallarchaeia* clades (Fig. 3A, Supplementary Dataset 5). Notably, almost all of the necessary bacteriochlorophyll synthesis components were widely distributed in *Heimdallarchaeia* MAGs from vents (including H2.bin.2 and LC2), which suggests that *Heimdallarchaeia* clades may be able to synthesize bacteriochlorophyll (Fig. 3A, Supplementary Dataset 5).

Protochlorophyllide reductase (Por) is a key enzyme in bacteriochlorophyll synthesis that could catalyze the transition between divinyl protochlorophyllide and divinyl chlorophyllide a [65]. Total four homologs of Por coding gene were found from these 13 *Heimdallarchaeia* MAGs. Phylogenetic analysis revealed that Por homologs from cold seep and vent were clustered in a sister clade, respectively (Fig. 3B, Supplementary Dataset 5). Then Por proteins from *Heimdallarchaeia*, *Acidobacteria*, and *Rhodobacterales* were located in branch with photosynthetic organisms, including *Cyanobacteria*, eukaryotic Algae, and Plants (*Streptophytina*). Interestingly, the Por homologs identified from *Bathyarchaeia* [66] displayed a closer evolutionary relationship to that of *Chloroflexia*, *Rhodospirillales*, and *Chromatiales* (Fig. 3B). These might indicate that Por homologs in *Heimdallarchaeia* are more closely evolutionarily related to eukaryotes compared with Por proteins of bacterial origin.

Bacteriochlorophyll synthase (BCS) is capable of synthesizing bacteriochlorophyll a by esterification of bacteriochlorophyllide with phytyl diphosphate or geranylgeranyl diphosphate [29]. Digeranylgeranylglyceryl phosphate synthase (DGPS) might perform the similar function in archaea. Key enzymes of bacteriochlorophyll biosynthesis, including BCS, have been reported in *Thermoproteota* (*Crenarchaeota*) [29] and *Bathyarchaeia* [30], suggesting that bacteriochlorophyll may be a common molecule used by archaea to utilize light. We phylogenetically analyzed the evolutionary relationship between BCS and DGPS, and found that archaeal DGPS was located at the outer group of the tree, separated from the clade containing BCS in phototrophic bacteria and chlorophyll synthase in photosynthetic organisms (Fig. 3C, Supplementary Dataset 5). Four homologues of BCS in the present *Heimdallarchaeia* MAGs clustered in a clade with the DGPS in *Heimdallarchaeia* LC2, which is located between the DGPS and BCS branches (Fig. 3C, Supplementary Dataset 5). Finally, a previously reported functional bacteriochlorophyll synthase derived from uncultured *Thermoproteota* (*Crenarchaeota*) [29] was found to cluster on a branch with the DGPS from *Heimdallarchaeia* LC3 [4]. This cluster displayed a close evolutionary relationship with the photosynthetic bacteriochlorophyll and chlorophyll synthase branches (Fig. 3C).

In addition to bacteriochlorophyll, other light-sensing pigments, including carotenoids [67, 68] and bacteriophytochrome [69], are identified to be synthesized in *Heimdallarchaeia* (Supplementary Dataset 6). Carotenoids are ubiquitous and essential pigments for photosynthesis [67]. Carotenoids function as accessory light-harvesting pigments that transfer absorbed energy to bacteriochlorophylls and thereby expand the range of wavelengths that are able to drive photosynthesis [70, 71]. We reconstructed the complete synthesis pathway of lycopene [72], a biologically important carotenoid derived from acetyl-CoA, using *Heimdallarchaeia* MAGs from cold seeps (Figure S5, Supplementary Dataset 6). It has been previously observed that light in the 450–550 nm (blue-green light) region of the solar radiation spectrum is not effectively absorbed by chlorophylls in photosynthesis, but is effectively absorbed by carotenoids [67, 68]. Moreover, carotenoids protect organisms from photo damage by quenching both singlet and triplet states of bacteriochlorophylls under strong illumination, and function as photosynthetic membrane stabilizers in chloroplasts [67]. Therefore, the biosynthesis of carotenoids in *Heimdallarchaeia* could complement bacteriochlorophyll to enable high-efficiency light energy utilization and thus provide a competitive advantage in habitats with light. What remains unclear is the ecological function or benefit of light energy utilization in *Heimdallarchaeia* clades, which reside predominantly in marine sediments [4].

A recent study on rhodopsins in *Heimdallarchaeia* provides evidence for their existence in light-exposed habitats that would provide sufficient energy [10]. The recovery of *Heimdallarchaeia* from deeper environments may be due to the high deposition rates characteristic of the sampling locations^10^. There is substantial evidence to demonstrate that both long wavelength (> 650 nm) and short wavelength (< 650 nm) light have been detected in vents [73, 74]. The blue-green light (450–550 nm) from the dim sunlight penetrating ocean water or the bioluminescence might exist in deep seafloor about 1,000 m [75, 76]. Our sampling site from the cold seep was also around this depth [77]. Thus, the necessary conditions for light energy utilization may exist in these environments. *Heimdallarchaeia* have the inferred capability to detect light of different wavelengths in the environment, and thus could utilize photoelectrons for energy conversion and thereby have an advantage in the competition for nutrient resources. However, more works are still needed to prove the functions of these pigments in *Heimdallarchaeia* for light sensing.

### The mixotrophic and aerobic lifestyle of *Heimdallarchaeia* clades

**Fig. 4:**
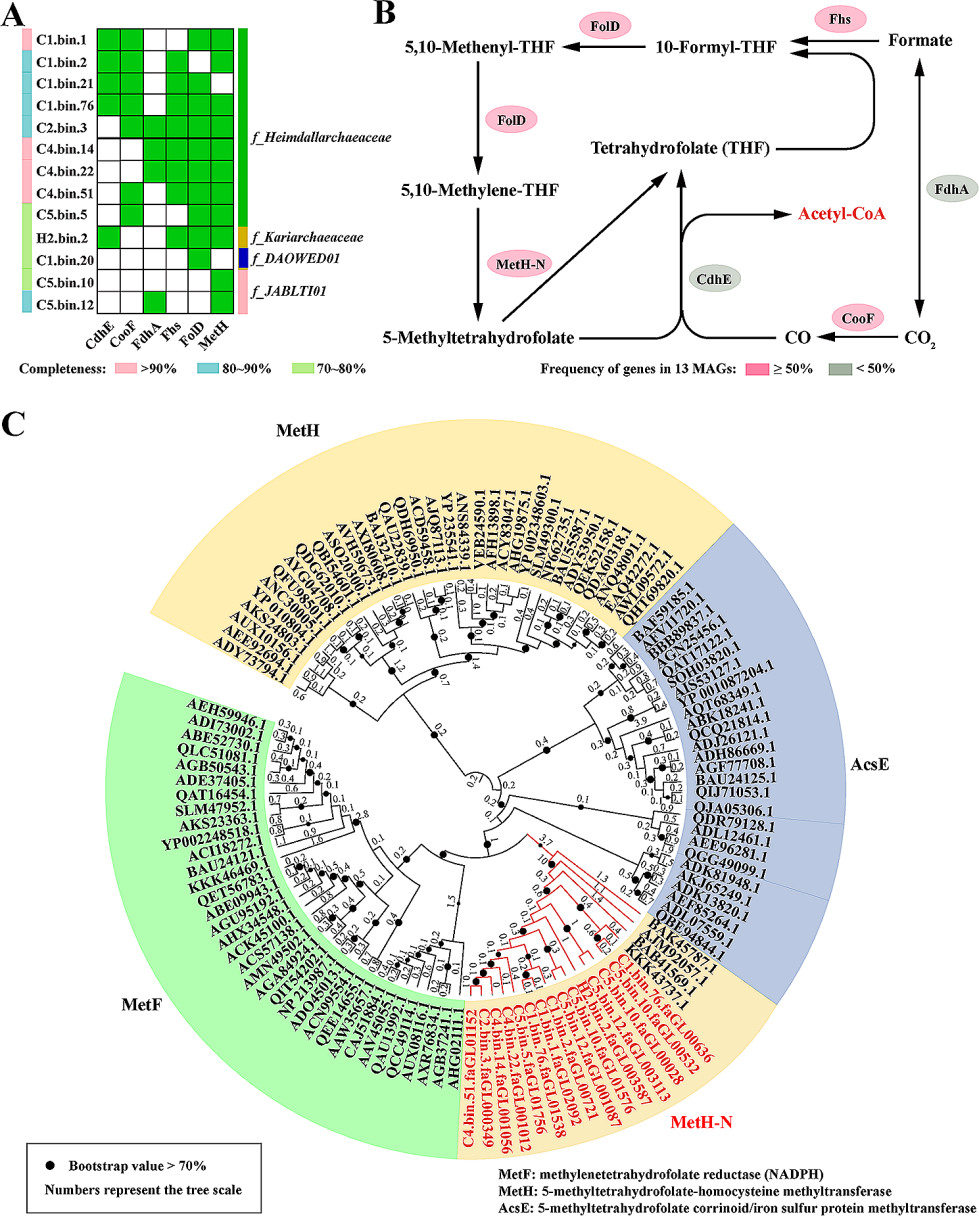
Wood-Ljungdahl (WL) pathway identified in *Heimdallarchaeia* clades. (**A**) Distribution of identified key enzymes involved in the WL pathway in *Heimdallarchaeia*. The presence of the enzymes involved in the WL pathway is indicated for each MAG using green colored rectangles. (**B**) WL pathway identified in *Heimdallarchaeia*. (**C**) Maximum-likelihood phylogeny of a potential difunctional enzyme (MetH) involved in the WL pathway in *Heimdallarchaeia* (using the LG + G4 + F model; 1,000 bootstrap replicates). Nodes indicate bootstrap values greater than 70. The numbers represent the tree scale of each branch. MetF, methylenetetrahydrofolate reductase; AcsE, 5-methyltetrahydrofolate corrinoid/iron sulfur protein methyltransferase; MetH, 5-methyltetrahydrofolate-homocystenie methyltransferase; CdhE (AscC), acetyl-CoA decarbonylase/synthase, CODH/ACS complex subunit gamma; CooF, anaerobic carbon-monoxide dehydrogenase iron sulfur subunit; FdhA, formate dehydrogenase (NADP^+^) alpha subunit; Fhs, formate-tetrahydrofolate ligase; FolD, methylenetetrahydrofolate dehydrogenase (NADP^+^)/methenyltetrahydrofolate cyclohydrolase. Detailed protein information related to this figure is listed in Supplementary Dataset 7

According to previous studies, *Heimdallarchaeia* clades exhibited a mixotrophic lifestyle similar to other members of the *Asgardarchaeota* MAGs [10, 11]. *Heimdallarchaeia* clades were able to simultaneously use the nearly complete TCA (from 2-oxoglutarate to malate) and transport exogenous organic matter through the metabolic circuitry for coupling catabolism with pyruvate metabolism [4, 11, 78]. They could also utilize a reverse tricarboxylic acid cycle (rTCA) for autotrophic CO_2_ assimilation [4, 10, 78]. In the present study, we found that these *Heimdallarchaeia* have the potential ability to fix CO_2_ with an atypical Wood-Ljungdahl pathway (Fig. 4A, Supplementary Dataset 7). In this atypical Wood-Ljungdahl process, the methylenetetrahydrofolate reductase (MetF) and 5-methyltetrahydrofolate corrinoid/iron sulfur protein methyltransferase (AcsE) are missing. However, a kind of protein (annotated as the bifunctional homocysteine S-methyltransferase/5,10-methylenetetrahydrofolate reductase, MetH) was found in deep-sea *Heimdallarchaeia* clades, which was not clustered with the traditional MetH proteins in the phylogenetic tree but located between the clades of AcsE and MetF. This novel function MetH protein (MetH-N) is thought to perform the functions of MetF and AcsE simultaneously, thereby catalyzing the production of tetrahydrofolate (THF) from 5,10-Methylene-THF (Fig. 4B, Supplementary Dataset 7) [79, 80]. In addition, CAZy (Carbohydrate-Active enZYmes database) analysis revealed a variety of polysaccharide-degrading enzymes, including chitinase, xylan/chitin deacetylase, diacetylchitobiose deacetylase, and cellulase, in these *Heimdallarchaeia* MAGs (Figure S6, Supplementary Dataset 6). These CAZymes and results previously presented suggest that macromolecular organic carbon compounds utilization and (homo)acetogenic fermentation may be the main metabolic strategies used for energy production by *Heimdallarchaeia* clades in deep-sea sediments, as shown for many other microorganisms [81]. Together, these versatile carbon metabolism patterns provide evidence that *Heimdallarchaeia* clades live a mixotrophic lifestyle which may be advantageous in deep-sea conditions [10].

**Fig. 5:**
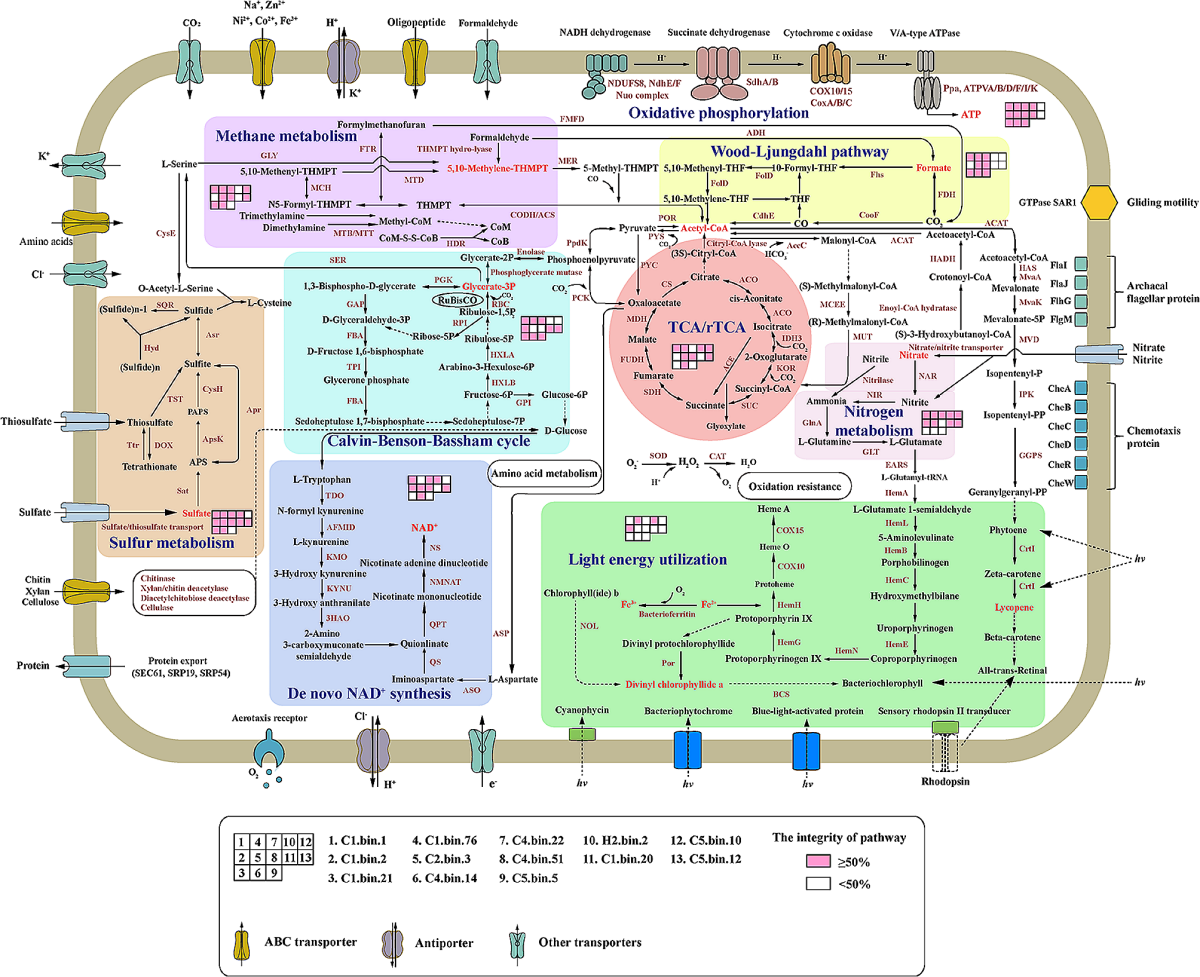
Reconstruction of the mixotrophic lifestyle of *Heimdallarchaeia*. Solid arrows indicate the enzymes associated with corresponding metabolic pathways identified in *Heimdallarchaeia* MAGs. Dotted arrows indicate the enzymes associated with corresponding metabolic pathways not identified in 13 MAGs in this study but in other *Heimdallarchaeia* MAGs, or some metabolic pathways different from the classical pathways in KEGG. Detailed information of key enzymes related to this figure is listed in Supplementary Dataset 6

Some aerobic metabolic pathways, distinguished from the metabolic pathways of anaerobic *Lokiarchaeia* and *Thorarchaeia* [4, 9–11], have been found in *Heimdallarchaeia* [9, 10]. We asked whether deep-sea *Heimdallarchaeia* clades could have a strict anaerobic lifestyle, given the surrounding environment. Similarly, aerobic respiration pathways such as the TCA and oxidative phosphorylation pathway were also found in deep-sea *Heimdallarchaeia* (Fig. 5, Supplementary Dataset 6). Moreover, both the aerobic kynurenine pathway and aspartate pathway for NAD^+ ^*de novo* synthesis were reconstructed in the present and previously published *Heimdallarchaeia* MAGs [10, 82]. In addition, other proteins involved in oxygen-dependent metabolism and peroxide removal, such as aerotaxis receptors, bacterioferritin, superoxide dismutase (SOD) and catalase (CAT), were identified in MAGs of deep-sea *Heimdallarchaeia* clades (Fig. 5). According to concentrations of dissolved oxygen (DO) in the South China Sea cold seep, there is more than 3 mg/L DO in the surface reduced sediments [77]. In the present study, the relative proportion of functional genes from *Heimdallarchaeia* in surface sediments (C1 and C2) is higher than that in deep sediments (C5), indicating that the distribution of *Heimdallarchaeia* is positively correlated with the concentration of DO (Figure S2). Taken together, this evidence strongly suggests that oxygen is present in the environments inhabited by *Heimdallarchaeia*, which seems to contradict the strict anaerobic lifestyle of other *Asgardarchaeota* (e.g. *Thorarchaeia* and *Lokiarchaeia*) that occupy the same habitat [4, 10]. We speculate this should be a survival strategy for *Heimdallarchaeia* to adapt to different environments. When living in the microaerobic environment, these *Heimdallarchaeia* could resist the toxicity of oxygen and use oxygen for physiological metabolism. However, *Heimdallarchaeia* could also transfer electrons through anaerobic respiration to obtain energy in the strict anaerobic environments.

## Conclusion

We analyze 13 MAGs of *Heimdallarchaeia* from the deep-sea cold seep and hydrothermal vent. We show that these *Heimdallarchaeia* clades enable reduce sulfate and nitrate to sulfide and ammonia, respectively, revealing their undiscovered roles in biogeochemical cycling of deep sea. Of note, we demonstrate that *Heimdallarchaeia* clades could synthesize bacteriochlorophyll and carotenoid, and might utilize light through these light-sensing pigments. In addition, we further found that *Heimdallarchaeia* clades could fix CO_2_ through an atypical Wood-Ljungdahl process, and a novel multi-function MetH enzyme might play a key role in this process. Lastly, we thus propose that *Heimdallarchaeia* possess a mixotrophic lifestyle, which may give them more flexibility to adapt to harsh deep-sea conditions, and make them contribute to the biogeochemical cycling in deep biosphere.

## Materials and methods

### Sample collection and processing

Samples were collected from cold seeps in the South China Sea and a hydrothermal vent field in the Western Pacific Trough (Table S1) during the cruise of the R/V *Kexue* in July 2018. Cold seep sediment samples (C1, C4, C2, and C5) were collected at depth intervals of 0–20, 20–40, 40–60 and 280 cm. The vent subsurface (0–20 cm) sediment sample (H2) was taken at the outside of the “black chimney” of the vent. Samples C1 and H2 were collected by the Discovery remotely operated vehicle (ROV), sample C4 was collected by the television grab, and samples C2 and C5 were collected by the gravity sampler. Sediments were sealed into sterile sampling bags immediately after collection and stored at -80 °C. DNA for metagenomics analysis was isolated from 20 g (wet weight) sediment following sample with the PowerSoil DNA Isolation Kit (Qiagen) per the manufacturer’s instructions.

### Analyses of environmental and chemical parameters of sampling sites

The temperature, salinity, and underwater depth of sampling sites were recorded in real time by an SBE 25plus Sealogger CTD (SBE, USA). Concentrations of CO_2_ and CH_4_ in surface sediments were measured in situ with CONTROS®HydroCO_2_ (CONTROS, Norway) and Hydro®CH_4_ (CONTROS, Norway) sensors, respectively. All sensors were mounted on the Discovery ROV. For chemical element analyses, all sediment samples (C1, C2, C4, C5, and H2) were dehydrated in an oven at 80 °C until completely dry. After grinding, sample powder was filtered through a 200-mesh screen. The filtrate was analyzed for chemical content, including Na, Mg, Fe, Cl, S, P, Mn, Zn, Ni, and Co, using an S8 Tiger X-ray fluorescence spectrometer (BRUKER, Germany).

### Library construction and sequencing

DNA extracts were treated with DNase-free RNase to eliminate RNA contamination, and DNA concentration was measured by a Qubit 3.0 fluorimeter (Thermo Fisher Scientific, USA). DNA integrity was checked by gel electrophoresis. 0.5 µg of each sample was used to prepare libraries. DNA was sheared into fragments between 50 and 800 bp in length using a Covaris E220 ultrasonicator (Covaris, UK); fragments between 150 bp and 250 bp were selected using AMPure XP beads (Agencourt, USA) and repaired using T4 DNA polymerase (ENZYMATICS, USA). DNA fragments were ligated at both ends to T-tailed adapters and amplified for eight cycles. Finally, the amplification products were used to produce single-stranded, circular DNA libraries. All NGS libraries were sequenced on a BGISEQ-500 platform (BGI, China) to obtain 100 bp paired-end raw reads. Quality control was performed by SOAPnuke (v1.5.6) (setting: -l 20 -q 0.2 -n 0.05 -Q 2 -d -c 0–5 0–7 1) [83].

### Genome assembly, binning and annotation

The raw shotgun sequencing metagenomic reads were dereplicated and trimmed using BGI-Qingdao (BGI, China). Clean data were assembled using MEGAHIT (v1.1.3, setting: --min-count 2 --k-min 33 --k-max 83 --k-step 10) [84]. Thereafter, metaBAT2 [85], Maxbin2 [86] and Concoct [87] were used to automatically bin from assemblies. Finally, MetaWRAP [88] was used to purify and generate data to obtain the final bins. Manual curation was adapted to reduce genome contamination based on differential coverage, GC content, and the presence of duplicate genes. The completeness and contamination of the genomes within bins were then estimated using CheckM [89]. Gene prediction for individual genomes was performed using Glimmer (v 3.02) [90]. The KEGG (Kyoto Encyclopedia of Genes and Genomes, Release 87.0), NR (Non-Redundant Protein Database databases, 20,180,814), Swiss-Prot (release-2017_07) and EggNOG (2015-10_4.5v) databases were used to annotate protein functions by default (cutoff E-values < 1E-5), and the best hits were chosen. The domain of these proteins from different MAGs were further aligned by Pfam (37.0, Pfam-A.hmm) with the cutoff E-values < 1E-20. Additionally, the CAZy (Carbohydrate-Active enZYmes) [91] database was downloaded to search for carbohydrate active enzymes within genomic bins. Sulfur metabolism related proteins were searched by HMMER (3.3.1) with the function of hmmscan [92].

### Phylogenetic analyses

To reveal the phylogenetic relationships between the assembled Archaea genomes, the 450 genomic sequences of Archaea were downloaded from GTDB genome databases using Aspera (v3.9.8), and 53 marker proteins (Dataset S1) were chosen from GTDB database (release 220) with automated settings. The concatenated sequences were trimmed with TrimAl (version 1.2) [93] using the gappyout function. Finally, the maximum-likelihood tree was calculated using IQ-TREE (v1.6.12) [94] with the GTR + F + I + G4 model and 1,000 bootstrap replicates (-bb 1,000).

To assess the type of SQRs in MAGs of *Heimdallarchaeia*, homologous amino acid sequences of SQR [41, 43], were extracted from NCBI databases (including type I to type VI SQRs). After sequence alignment, a maximum-likelihood phylogenetic tree was constructed with the LG + I + G4 + F model (-bb 1,000) using IQ-TREE.

Full-length nitrilase protein sequences were selected from NCBI databases according to the classification of their inducers (e.g. aliphatic nitriles, aromatic nitriles, and amides). Nitrilase homologs mainly included nitrilases derived from archaea, bacteria, and fungi. Sequences of nitrilases from MAGs of *Heimdallarchaeia* were aligned and processed through maximum-likelihood phylogenetic analyses using IQ-TREE with the LG + G4 + F model (-bb 1,000).

Phylogenetic analyses were performed to explain the relationship between newly discovered bifunctional catalyzing enzymes in MAGs of *Heimdallarchaeia* and classical catalyzing enzymes in archaea and bacteria, which involve in the conversion of 5,10-methylenetetrahydrofolate to tetrahydrofolate. Protein sequences for MetF and AcsE (from the Wood-Ljungdahl pathway) were selected from the KEGG database. In addition, protein sequences of MetH were downloaded to construct a maximum-likelihood phylogenetic tree with the LG + G4 + F model (-bb 1,000) using IQ-TREE.

To analyze the evolutionary relationships between protochlorophyllide reductase (Por) and bacteriochlorophyll synthase (BCS) in MAGs of *Heimdallarchaeia* and reported photosynthetic organisms, homologous sequences were selected from archaea, bacteria, and eukaryotes in NCBI and Swiss-Prot databases. The aligned sequences were constructed using maximum-likelihood phylogenetic analyses in IQ-TREE with the WAG + G4 and LG + F + I + G4 models (-bb 1,000), respectively. The sequence alignments for all trees were calculated using the MEGA X soft with Clustal W/MUSCLE program [95]. All trees were visualized using iTOL (v5) [96].

CompareM (v 0.0.23) with aai_wf function [97] was used to calculate the average amino acid identity (AAI) across all MAGs and new phyla derived from *Heimdallarchaeia* referenced genomes. Results were displayed as a heatmap using R (3.5.1).

### Electronic supplementary material

Below is the link to the electronic supplementary material.

Supplementary Material 1

Supplementary Material 2

Supplementary Material 3

Supplementary Material 4

Supplementary Material 5

Supplementary Material 6

Supplementary Material 7

Supplementary Material 8

Supplementary Material 9

## Data Availability

The Heimdallarchaeia MAGs (C1.bin.1, C1.bin.2, C1.bin.20, C1.bin.21, C1.bin.76, C2.bin.3, C4.bin.14, C4.bin.22, C4.bin.51, C5.bin.5, C5.bin.10, C5.bin.12 and H2.bin.2) obtained in this study are available in NCBI Genbank under the accession numbers SAMN15815161, SAMN15815162, SAMN15815163, SAMN15815164, SAMN15815165, SAMN13483368, SAMN13483369, SAMN13483392, SAMN15815166, SAMN15815166, SAMN15815168, SAMN13483370, and SAMN13483372, respectively, in BioProject PRJNA593668.
