# Supplementary material for: Metagenomic insights into Heimdallarchaeia clades from the deep-sea cold seep and hydrothermal vent
Source: Environ Microbiome. 2024 Jun 22;19:43. doi: 10.1186/s40793-024-00585-2 (PMC11193907; doi:10.1186/s40793-024-00585-2)
Supplement: Supplementary file 8 — Supplementary Material 8 [file 40793_2024_585_MOESM8_ESM.docx]

**Metagenomic insights into *Heimdallarchaeia* clades from the deep-sea cold seep and hydrothermal vent**

Rui Liu^1,2,5^, Ruining Cai^1,2,5^, Minxiao Wang^1,4,5^, Jing Zhang^1,2,5^, Huan Zhang^4,5^, Chaolun Li^3,4,5*^, Chaomin Sun^1,2,4,5*^

^1^CAS and Shandong Province Key Laboratory of Experimental Marine Biology, Institute of Oceanology, Chinese Academy of Sciences, Qingdao, China

^2^Laboratory for Marine Biology and Biotechnology, Qingdao National Laboratory for Marine Science and Technology, Qingdao, China

^3^Key Laboratory of Marine Ecology and Environmental Sciences, Institute of Oceanology, Chinese Academy of Sciences, Qingdao, China

^4^Center of Deep Sea Research, Institute of Oceanology, Chinese Academy of Sciences, Qingdao, China

^5^Center of Ocean Mega-Science, Chinese Academy of Sciences, Qingdao, China

^*^ Corresponding author

Chaomin Sun Tel.: +86 532 82898857; fax: +86 532 82898857.

E-mail address: sunchaomin@qdio.ac.cn

Chaolun Li E-mail address: lcl@qdio.ac.cn

**Table S1. The geographic positions of sampling sites**

| Sample name | Sample type | Location | Longitude and latitude | Depth under the surface of ocean (m) | Depth under the surface of sediment (cm) |
| --- | --- | --- | --- | --- | --- |
| C1 | Sediment | Cold seep | E 119º17'07.106''  N 22º06'55.114'' | 1146 | 0~20 |
| C4 | Sediment | Cold seep | E 119º17'07.322''  N 22º06'58.598'' | 1121 | 20~40 |
| C2 | Sediment | Cold seep | E 119º17'06.436''  N 22º06'55.169'' | 1137 | 40~60 |
| C5 | Sediment | Cold seep | E 119º17'06.436''  N 22º06'55.169'' | 1137 | 280~300 |
| H2 | Sediment | Hydrothermal vent | E 124º22'23.374''  N 25º15'49.868'' | 2194 | 0~20 |

**Table S2. Assembly statistics and quality metrics of reconstructed *Heimdallarchaeia* MAGs**

| Bin name | Completeness (%) | Contamination (%) | GC (%) | N50 (bp) | Genome size (Mbp) | Number of genes |
| --- | --- | --- | --- | --- | --- | --- |
| C1.bin.1 | 92.52 | 1.432 | 33.42 | 5375 | 3.11 | 2618 |
| C1.bin.2 | 82.67 | 2.342 | 32.88 | 13723 | 2.75 | 2128 |
| C1.bin.20 | 73.02 | 1.867 | 33.56 | 4134 | 3.04 | 2010 |
| C1.bin.21 | 85.51 | 0.931 | 33.38 | 6299 | 3.22 | 2591 |
| C1.bin.76 | 85.51 | 0.000 | 33.46 | 17300 | 3.02 | 2411 |
| C2.bin.3 | 86.84 | 5.607 | 33.50 | 10642 | 3.06 | 2885 |
| C4.bin.14 | 91.58 | 3.738 | 33.40 | 41762 | 3.27 | 3084 |
| C4.bin.22 | 92.52 | 3.738 | 34.20 | 25712 | 2.82 | 2593 |
| C4.bin.51 | 91.59 | 4.213 | 33.20 | 10485 | 3.91 | 3306 |
| C5.bin.5 | 71.44 | 2.817 | 33.85 | 2839 | 2.88 | 2006 |
| C5.bin.10 | 75.46 | 3.740 | 33.93 | 10474 | 2.89 | 2013 |
| C5.bin.12 | 80.84 | 6.540 | 33.80 | 11218 | 3.24 | 3128 |
| H2.bin.2 | 78.03 | 5.295 | 34.30 | 15481 | 4.85 | 4450 |

**
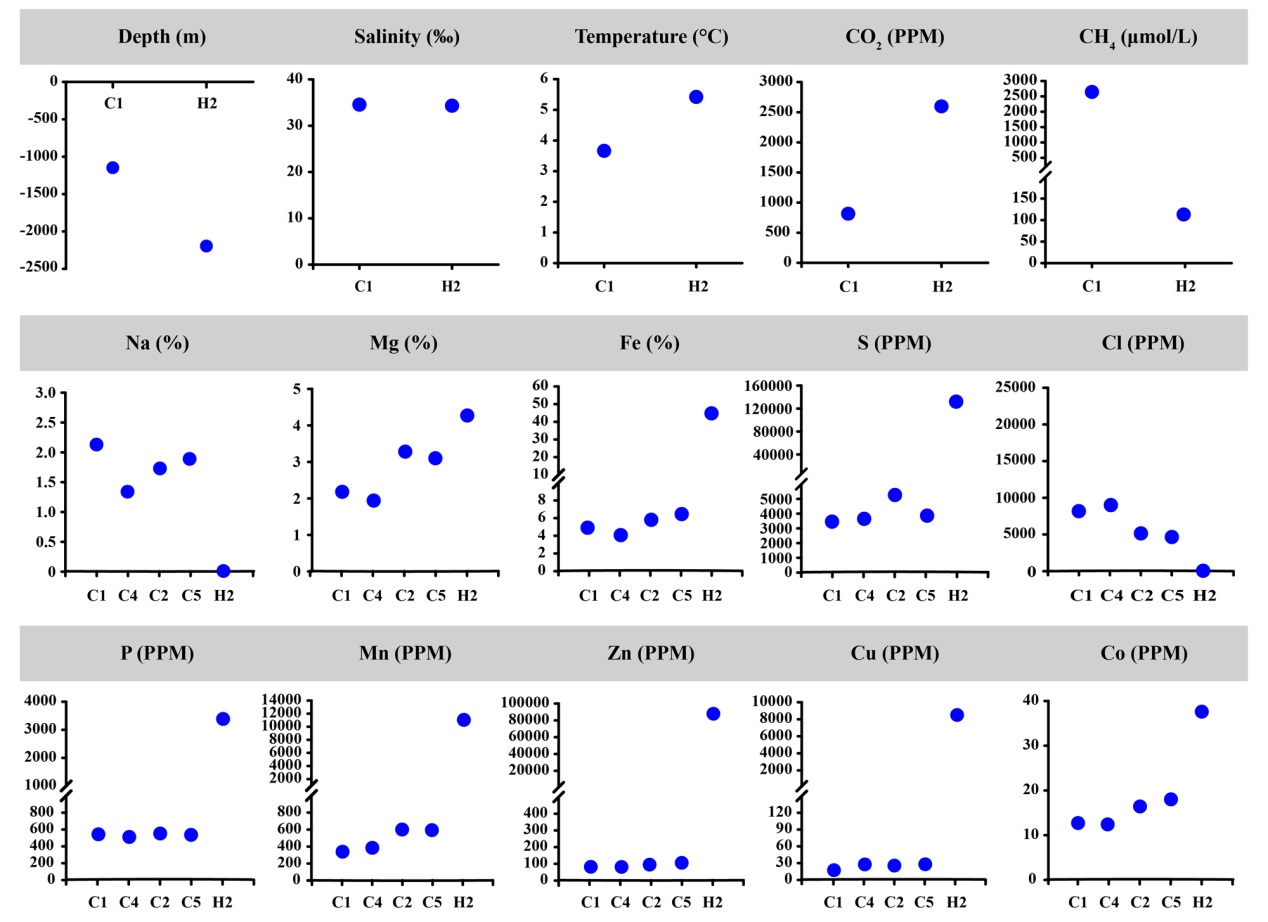
**

**Fig. S1.** Analyses of environmental and chemical parameters of sampling sites in the deep-sea cold seeps and hydrothermal vent. The temperature, salinity, underwater depth were recorded in real-time by SBE 25plus Sealogger CTD, and concentrations of CO_2_ and CH_4_ of surface sediments were measured *in situ* with the CONTROS®HydroCO_2_ and Hydro®CH_4_. Contents of different elements including Na, Mg, Fe, Cl, S, P, Mn, Zn, Ni and Co were measured by an S8 Tiger X-ray fluorescence spectrometry.


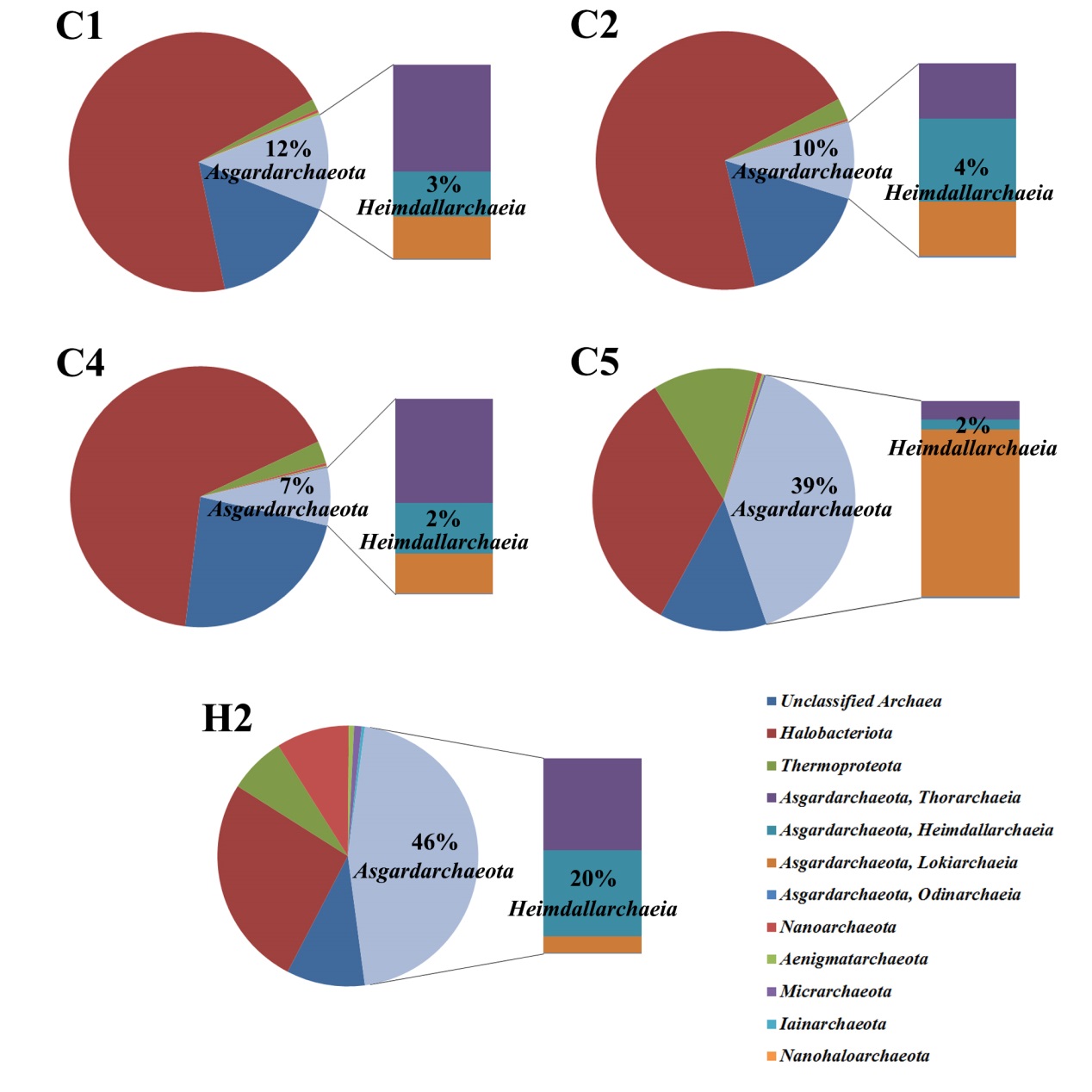


**Fig. S2.** Ratios of annotated genes derived from *Asgardarchaeota* to the whole Archaeal metagenomic genes in the five samples (C1, C2, C4, C5 and H2) used in this study.


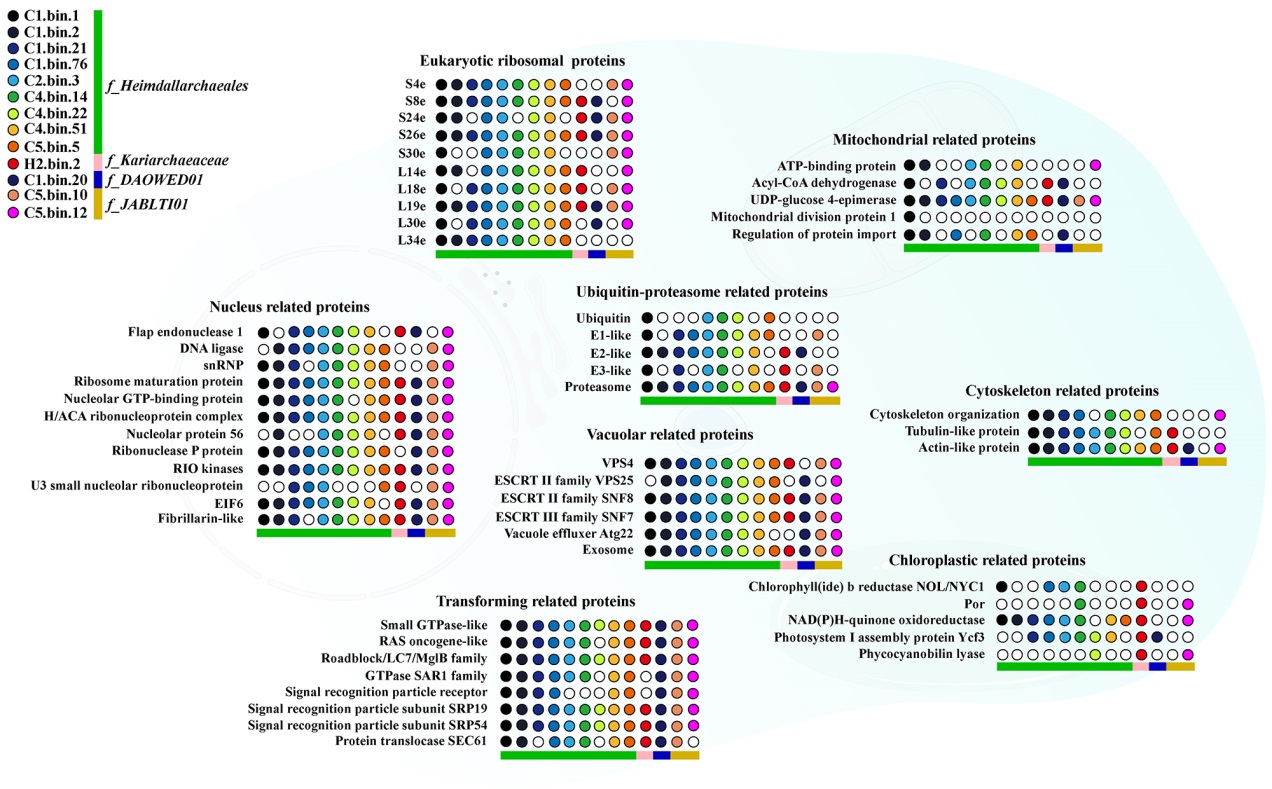


**Fig. S3.** Eukaryotic signatures identified in *Heimdallarchaeia* clades. Schematic representation of a eukaryotic-like cell highlighting which eukaryote-specific proteins have been identified in MAGs of *Heimdallarchaeia*. *Heimdallarchaeia* clades contain both reported eukaryotic signatures and unprecedented chloroplastic related proteins. Detailed protein information related to this figure is listed in Supplementary Dataset 2.

**
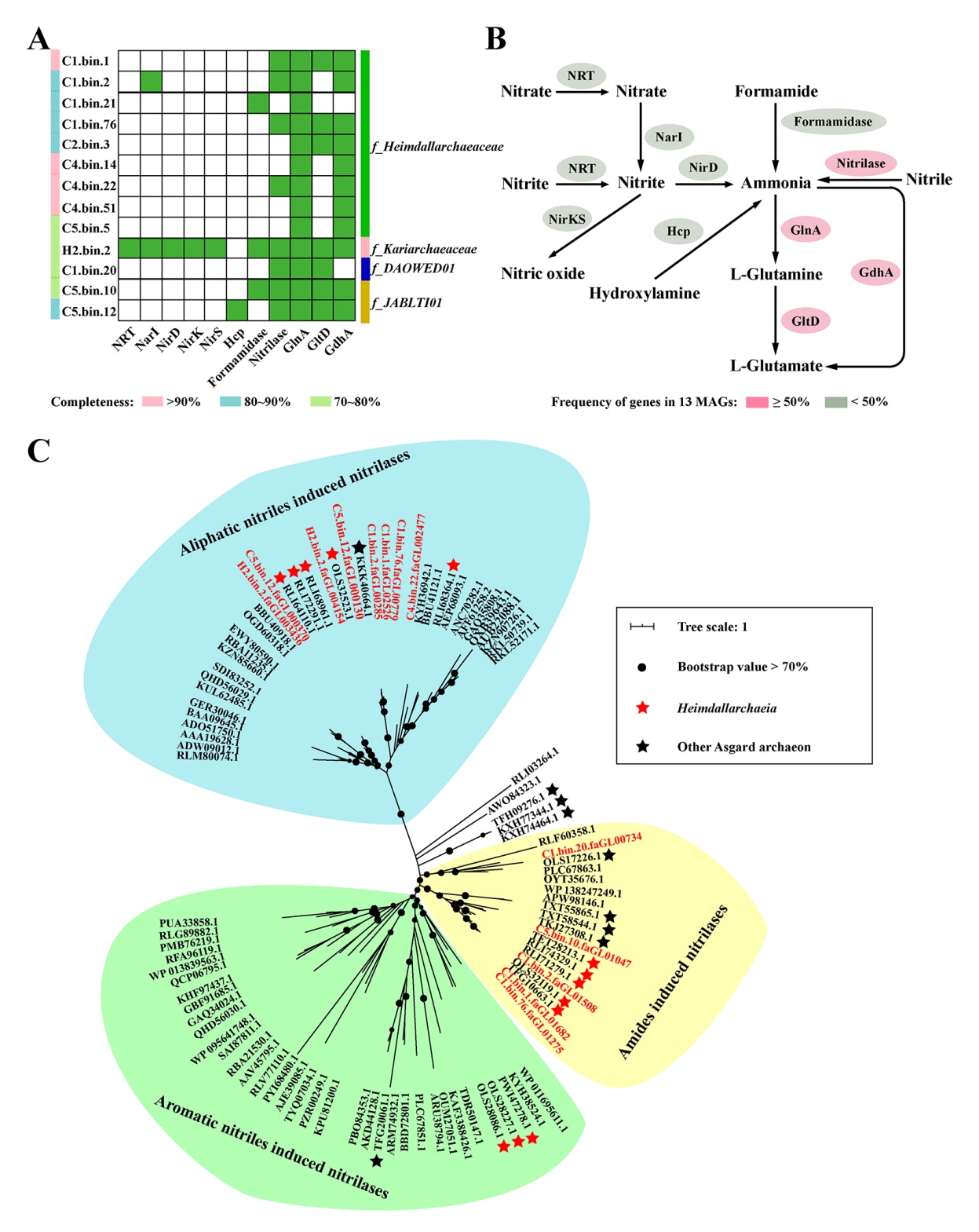
**

**Fig. S4.** Nitrogen metabolic pathway identified in *Heimdallarchaeia* MAGs. (A) The distribution of identified key enzymes involved in nitrogen metabolism of *Heimdallarchaeia*. The presence of the enzymes involved in the nitrogen metabolic pathway is indicated for each MAG using green colored rectangles. (B) Nitrogen metabolic pathway identified in *Heimdallarchaeia*. (C) Maximum-likelihood phylogeny of nitrilase in *Heimdallarchaeia* with LG+G4+F model (1,000 bootstrap replicates). The nodes are shown as bootstrap values greater than 70. The red and black pentacles indicate the nitrilase identified in the present and previous *Heimdallarchaeia* MAGs, respectively. NRT, nitrate/nitrite transporter; NarI, Nitrate reductase gamma subunit; NirD, nitrite reductase (NADH) small subunit; NirK, nitrite reductase (NO-forming); Hcp, hydroxylamine reductase; GlnA, glutamine synthetase; GltD, glutamate synthase (NADPH) small chain; GdhA, glutamate dehydrogenase (NADP^+^). All detailed protein information mentioned in this figure is listed in the Supplementary Dataset 4.


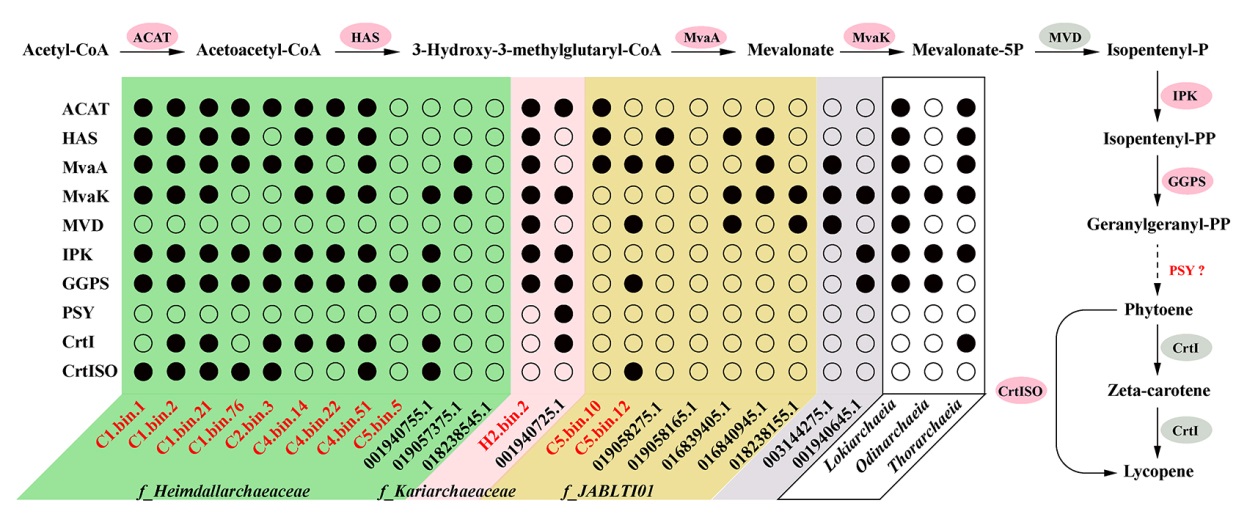


**Fig. S5.** Carotenoid biosynthesis pathway identified in *Heimdallarchaeia* MAGs. The solid arrows indicate the enzymes associated with carotenoid biosynthesis present in *Heimdallarchaeia* MAGs. Dotted arrows indicate the enzymes associated with carotenoid biosynthesis absent in MAGs. The gray box highlights MAGs from *o*_*Hadarchaeales*. The frame highlights assembled genomes of other *Asgardarchaeota*. The red highlights MAGs obtained in this study. ACAT, acetoacetyl-CoA thiolase; HAS, hydroxymethylglutaryl-CoA synthase; MvaA, hydroxymethylglutaryl-CoA reductase; MvaK, mevalonate kinase; MVD, diphosphomevalonate decarboxylase; IPK, isopentenyl phosphate kinase; GGPS, geranylgeranyl diphosphate synthase; CrtI, phytoene desaturase; CrtISO, all-trans-zeta-carotene desaturase. The detailed information of key enzymes involved in carotenoid biosynthesis is listed in the Supplementary Dataset 7.

**
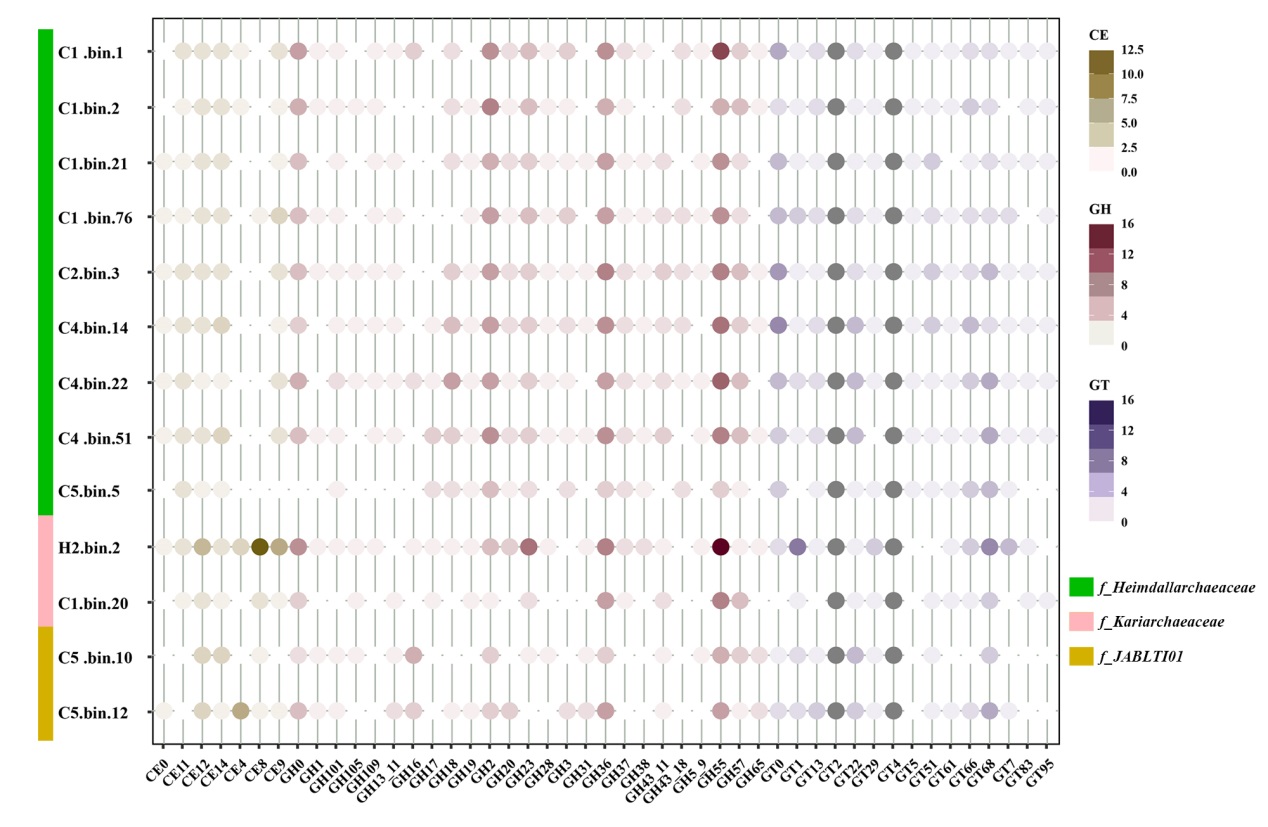
**

**Fig. S6.** The carbohydrate degradation enzymes identified in *Heimdallarchaeia* MAGs by CAZy analysis. CE, carbohydrate esterase. GH, glycoside hydrolase. GT, glycosyltransferase. All detailed protein information mentioned in this figure is listed in the Supplementary Dataset 6.
